# Nasal high-flow oxygen in patients with hypoxic respiratory failure: effect on functional and subjective respiratory parameters compared to conventional oxygen therapy and noninvasive ventilation

**DOI:** 10.1186/cc10743

**Published:** 2012-03-20

**Authors:** R Riessen, N Schwabbauer, B Berg, G Blumenstock, M Haap, J Hetzel

**Affiliations:** 1 University Hospital Tübingen, Germany

## Introduction

This study compared a nasal high-flow oxygen therapy (NHFO_2_) with conventional oxygen therapy via a Venturi mask (VM) or noninvasive ventilation (NIV) in patients with hypoxic respiratory failure. Study endpoints were functional respiratory parameters, dyspnea, patient comfort and a global rating by the patients.

## Methods

We included 14 patients with hypoxic respiratory failure (paO_2 _<55 mmHg under room air). Exclusion criteria were ventilatory failure, hemodynamic instability, cardiogenic pulmonary edema, NIV contraindications and inability to cooperate. Patients were treated in a randomized order for 30 minutes each with NHFO_2 _(Optiflow^®^; Fisher-Paykel), VM or NIV, using a FiO_2 _of 0.6. Every treatment phase was preceded by a 15-minute baseline phase in which the patients received oxygen via a standard nasal prong (SaO_2 _goal >88%). At the end of each treatment phase vital signs and blood gases were measured and patients rated their dyspnea and their general comfort on a 10-point scale. Finally, patients were ask for a global rating of all three devices ranging from 1 (very good) to 6 (failed) and could choose one device for further treatment.

## Results

The paO_2 _was highest under NIV with 129 ± 38 mmHg, followed by NHFO_2 _(101 ± 34 mmHg, *P *< 0.01 vs. NIV) and VM (85 ± 21 mmHg, *P *< 0.001 vs. NIV, *P *< 0.01 vs. NHFO_2_, ANOVA). All other vital and blood gas parameters did not show significant differences. Dyspnea rating on a 10-point Borg scale was significantly better under NHFO_2 _(2.9 ± 2.1) and VM (3.3 ± 2.3) compared to NIV (5.0 ± 3.3) (*P *< 0.05, vs. NHFO_2 _or VM). Comfort rating showed similar results: NHFO_2 _2.7 ± 1.8; VM 3.1 ± 2.8; NIV 5.4 ± 3.1 (*P *< 0.05, NIV vs. NHFO_2 _or VM). In the final global rating using German school grades from 1 to 6 NHFO_2 _also received the best rating (2.3 ± 1.4), followed by VM (3.2 ± 1.7, *P *= NS vs. NHFO_2_) and NIV (4.5 ± 1.7, *P *< 0.01 vs. NHFO_2 _and *P *< 0.05 vs. VM). For further treatment 10 patients chose NHFO_2_, three VM and one NIV.

## Conclusion

NHFO_2 _is a promising new device for oxygen supply in respiratory failure, offering better oxygenation than the VM and better patient comfort and tolerance than NIV.

